# Serotonin system in the human placenta – the knowns and unknowns

**DOI:** 10.3389/fendo.2022.1061317

**Published:** 2022-12-01

**Authors:** Maja Perić, Ivona Bečeheli, Lipa Čičin-Šain, Gernot Desoye, Jasminka Štefulj

**Affiliations:** ^1^ Laboratory of Neurochemistry and Molecular Neurobiology, Division of Molecular Biology, Ruđer Bošković Institute, Zagreb, Croatia; ^2^ Department of Obstetrics and Gynecology, Medical University of Graz, Graz, Austria

**Keywords:** human placenta, pregnancy, serotonin, serotonin receptors, serotonin transporters, MAO, TPH, fetal sex

## Abstract

The biogenic monoamine serotonin (5-hydroxytryptamine, 5-HT) is a chemical messenger widely distributed in the brain and various other organs. Its homeostasis is maintained by the coordinated activity of a variety of proteins, including enzymes of serotonin metabolism, transmembrane transporters of serotonin, and serotonin receptors. The serotonin system has been identified also in the placenta in rodent models as a key component of placental physiology. However, serotonin pathways in the human placenta are far from well understood. Their alterations may have long-lasting consequences for the fetus that can manifest later in life. In this review, we summarize information on the location of the components of the serotonin system in the human placenta, their regulation, function, and alterations in pathological pregnancies. We highlight current controversies and discuss important topics for future research.

## 1 Introduction

The placenta is a temporary fetal organ that develops early in pregnancy. It acts as a barrier/facilitator for transport of gases, nutrients, and waste between maternal and fetal blood. In addition, the placenta produces and secretes a variety of hormones, cytokines, and growth factors that are essential for proper placental and fetal development and for facilitating maternal adaptation to and maintenance of pregnancy (1). Human placental development begins shortly after the blastocyst implants into the uterine wall, when cytotrophoblast cells begin to proliferate and fuse into a multinucleate syncytiotrophoblast. During the first trimester of pregnancy, as the cytotrophoblasts continue to invade the uterine wall, protrusions and fetal vessels form, eventually giving rise to chorionic villi. At the end of the first trimester, flow of fully oxygenated maternal blood is established enabling maternal blood to fill the lacunae formed within the syncytiotrophoblast (2). At the sites where the chorionic villi anchor into the maternal decidua (anchoring villi), a subpopulation of trophoblasts, the extravillous cytotrophoblasts, remodels the maternal spiral arteries by transiently partially replacing the endothelial cells lining the arteries and colonizing the inner layers of the myometrium (1, 3). Finally, the placental barrier formed consists of feto-placental endothelial cells lining the fetal capillaries, a mesenchymal core (Hofbauer cells, fibroblasts, and collagenous stroma), and cytotrophoblast cells overlaid by a continuous layer of syncytiotrophoblast (4). Although essential to the life of the fetus, the placenta is in many aspects still a rather mysterious organ.

Primary biogenic monoamines, such as dopamine, epinephrine, norepinephrine and serotonin, are widely used chemical messengers that act as neurotransmitters, hormones and autacoids. As low weight molecules with an amino group attached to an aromatic ring, all are synthesized from aromatic L-amino acids and inactivated by removal of the amino group. They play various regulatory roles in the nervous system and other organ systems. Serotonin not only regulates functions in the mature organism, but also acts as a significant growth factor during development and regulates various developmental processes, including nervous system development. Homeostasis of serotonin signaling during both development and adulthood is maintained by the coordinated activity of a variety of serotonin-regulating proteins, including its metabolic enzymes, membrane transporters and receptors. These proteins are widely distributed in the brain and various other organs, including the placenta.

Available evidence from clinical and animal studies suggests that placental serotonin system regulates placental development and functions and plays a role in proper embryo/fetal development. Its alterations may have long-lasting consequences for the fetus, which may manifest later in life like reverberations of the original effect. Despite the emerging evidence of the important role of serotonin for the feto-placental unit, its pathways in the human placenta are still far from being understood.

Here, we summarize the current data on the presence, regulation and function of serotonin system components in the human placenta, as well as on their putative role in normal and pathological pregnancy. We also highlight gaps and controversies in current knowledge and discuss directions for future research.

## 2 Serotonin system and its components in general 

Serotonin (5-hydroxytryptamine, 5-HT), originally called enteramine, is a biogenic monoamine first isolated in 1937 from enterochromaffin cells of the intestine, and shown to induce intestinal contractions (5). In the 1940s, the same substance was identified as a “tone-modifier” present in serum and therefore named serotonin (6). Soon thereafter, its synthesis was discovered in the brain and more recently in several other peripheral sites (cf. below).

Today, serotonin is best known for its neurotransmitter role in the brain, but it acts also as a hormone and autocrine/paracrine messenger in various other organs (7). Many of its roles are highly conserved in a variety of animal species (8). In vertebrates, it modulates brain functions such as mood, emotion, cognition, sleep/wake rhythm, appetite, sexual behavior, pain perception, and response to stress (9). In addition, it regulates and fine tunes numerous other physiological processes, including hemostasis and vascular tone, gastrointestinal functions, immune response, reproductive functions, bone remodeling, and energy balance (7, 10, 11). Moreover, it contributes to regulating organ development and regeneration, by controlling basic cellular processes such as proliferation, differentiation, and migration (12, 13). In the developing fetal brain, it acts as a neurotrophic factor for various neuronal populations and also regulates the maturation of its own neurons (14, 15). Less well known are the antioxidant effects of serotonin in scavenging reactive oxygen species and inhibiting lipid peroxidation (16).

The effects of serotonin are primarily mediated by its interaction with plasma membrane receptors. In humans, 14 different serotonin receptor subtypes were identified and classified into 7 families (HTR1 to HTR7) based on structural, pharmacological, and signal transduction properties. With the exception of HTR3, a ligand-gated cation channel, all other subtypes are G protein-coupled receptors that activate various intracellular signaling pathways (17, 18). Serotonin receptors are widely distributed in the brain and various peripheral organs. During development, several serotonin receptor subtypes emerge in the developing brain prior to enzymes for serotonin synthesis, suggesting an extra-embryonic source of serotonin in the early stages of neurodevelopment (19).

Serotonin can also act in a receptor-independent manner by covalently binding to glutamine residues of various extracellular, cytoplasmic or nuclear proteins, in a process known as serotonylation. Serotonylation has been implicated in the regulation of platelet functions (20, 21), insulin secretion (22) and gene expression regulation (23).

Most of serotonin in the human body is produced and stored in the gastrointestinal tract. Some of the intestine-derived serotonin is secreted into the blood, where it is rapidly internalized in platelets and only a tiny fraction (< 1%) remains free in plasma (24). There are other niches of local serotonin synthesis in the human body, including serotonergic neurons, pinealocytes, pulmonary artery endothelial cells, mammary epithelial cells, mast cells, pancreatic beta cells, adipocytes, hepatocytes, osteoclasts, melanocytes, keratinocytes, and fibroblasts (25–27).

**Figure 1:**
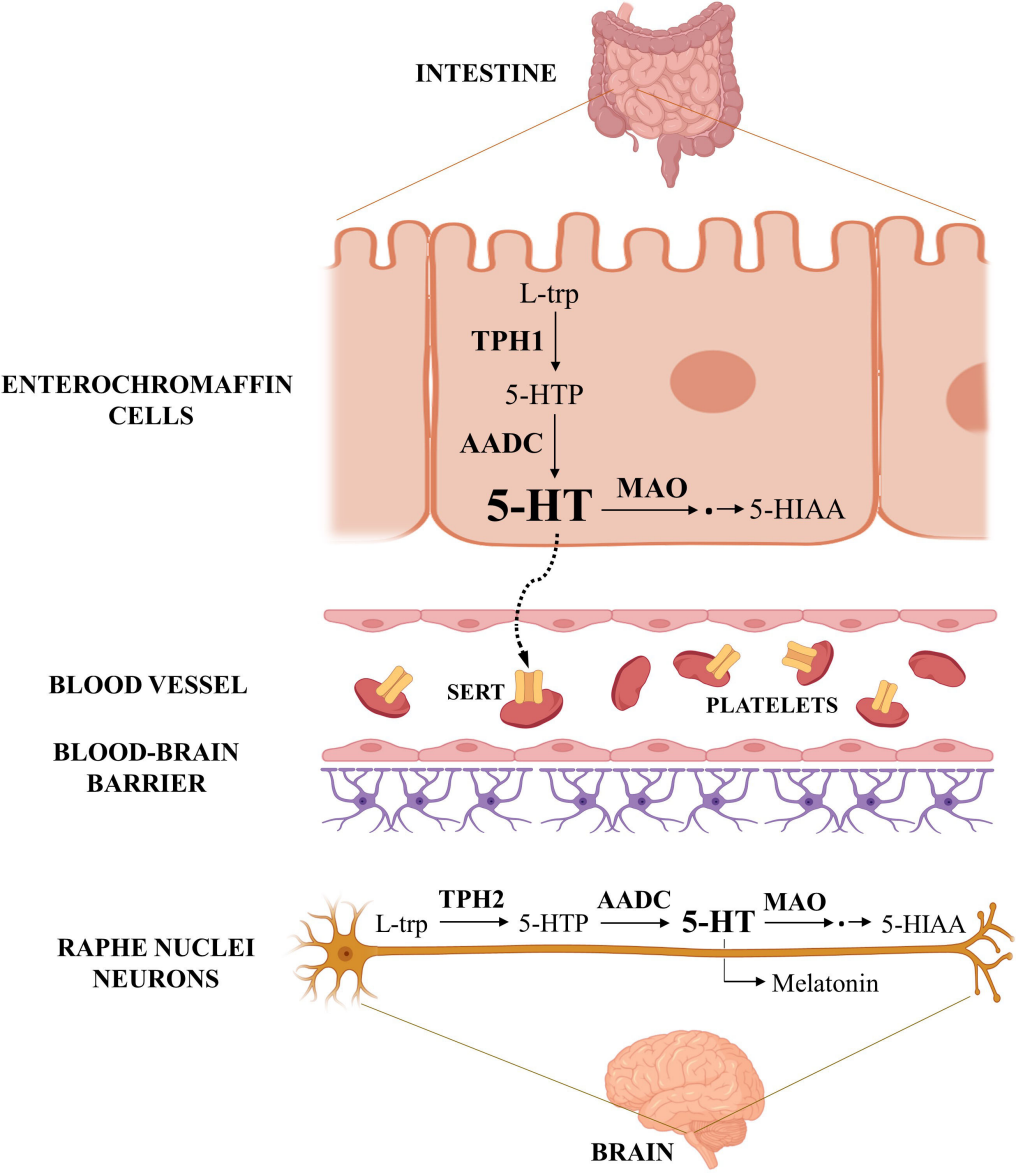
Serotonin metabolism. The figure shows the serotonin pathways in the gastrointestinal tract and in the central nervous system, the main sites of its synthesis in the human body. Serotonin synthesized in the enterochromaffin cells of the intestine is released into the portal circulation, taken up into platelets, and distributed to various other organs. Created with BioRender.com.

Serotonin biosynthesis uses essential amino acid L-tryptophan (L-trp) as precursor and involves two enzymatic steps (**Figure 1**). The first step, conversion of L-trp to 5-hydroxytryptophan (5-HTP), is catalyzed by rate-limiting tryptophan hydroxylase (TPH) and subsequent decarboxylation of 5-hydroxytryptophan to serotonin, is catalyzed by aromatic acid decarboxylase. There are two TPH isoforms, TPH1 and TPH2, encoded by genes located on human chromosome 11 and 12, respectively. The two TPH isoforms have different tissue expression – TPH1 (peripheral) is abundant in enterochromaffin cells of the intestine and in other peripheral tissues, whereas TPH2 (neuronal) is found mainly in serotonergic neurons of the brain (28).

Serotonin is catabolized mainly to the final metabolite 5-hydroxyindoleacetic acid (5-HIAA), which can be excreted by the kidneys. The first step in this catabolic pathway, oxidative deamination, is catalyzed by the outer mitochondrial membrane enzyme monoamine oxidase (MAO). Its two isoforms, MAOA and MAOB, are encoded by distinct genes on the human X chromosome (29). Both isoforms catalyze oxidative deamination of serotonin and various other endogenous and dietary monoamines, with MAOA having preferential affinity for 5-HT over other substrates (30). Serotonin can also be converted into melatonin *via* two enzymatic steps.

As a hydrophilic substance, serotonin cannot freely pass through the phospholipid bilayer of the plasma membranes but is transported by specialized transmembrane proteins. These plasma membrane transporters mediate the uptake of extracellular serotonin into cells, which is the key mechanism responsible for terminating receptor-mediated serotonin signaling. There are two plasma membrane transport systems for serotonin, with fundamentally different kinetic properties: the high-affinity/low capacity (uptake-1) system and the low-affinity/high capacity (uptake-2) system. The uptake-1 system is represented by the serotonin transporter (SERT, also known as 5-HTT) (31), whereas the uptake-2 system includes the plasma membrane monoamine transporter (PMAT) and organic cation transporters (OCTs) 1, 2 and 3 (32). SERT is highly selective for serotonin while PMAT and OCTs can transport both serotonin and other monoamines.

Once inside the cell, another group of transmembrane transporters, the vesicular monoamine transporters (VMATs), mediate the transfer of cytosolic serotonin into secretory/storage organelles such as dense granules in platelets, synaptic vesicles in neurons, and secretory granules in enterochromaffin cells, pancreatic beta cells, mastocytes, and adipocytes (33–35). The storage of serotonin in intracellular organelles protects it from degradation by MAO and enables its release *via* exocytosis. There are two closely related VMATs, VMAT1 and VMAT2, with different pharmacological properties and tissue distribution (36).

Export of serotonin from cells storing the amine in secretory/storage organelles occurs *via* calcium-stimulated exocytosis (37–39). The export mechanism(s) from cells where it is not stored in intracellular vesicles are much less well understood. One possible efflux pathway could be *via* OCT2 (40) or OCT3 (41), which transport organic cations in both directions across the plasma membrane. It has been observed that SERT also reverses the direction of transport in the presence of some exogenous substrates (42), but it is unknown whether this phenomenon occurs under physiological conditions. Serotonin “leakage” *via* passive diffusion is generally considered insignificant because of its hydrophilic properties, but ability of serotonin to bind to lipid membranes (43) suggests that this may be more important than assumed. Nevertheless, a thorough investigation of more efficient and regulated, carrier-mediated efflux mechanisms for serotonin is warranted.

The activity of serotonin metabolizing enzymes, receptors, and transmembrane transporters is regulated by multiple mechanisms. In general, transcription of serotonin-related genes is modulated by genetic (44, 45) and epigenetic factors (45–47) and transcripts are further processed by prominent, tissue-specific alternative splicing and RNA editing (48, 49). In addition, the activity of serotonin-related proteins is controlled by post-translational modifications such as palmitoylation, phosphorylation, glycosylation, serotonylation, and disulfide bond formation, as well as by membrane trafficking, cell-surface localization and interactions with other proteins (50–55).

An interesting feature of the serotonin system, observed in both rodents and humans, is the presence of sex differences in some of its components resulting in functional differences between males and females. In humans, sex differences have been found in the concentration of serotonin and its metabolite 5-HIAA (56, 57) and in the rate of serotonin synthesis (58) in the central nervous system. The function/density of SERT and certain serotonin receptors in the human brain also differs between men and women (59, 60). In rodent models, pharmacological (61, 62) or genetic (63) manipulation of the serotonergic system causes sex-dependent behavioral and biochemical changes.

Sex differences in the serotonin system occur early in development. Studies in animal models have shown that the developing serotonergic system of males and females is differentially affected by various prenatal and perinatal factors including the microbiome (64), maternal overnutrition (65), and traumatic experiences such as parental separation (66). In humans, prenatal exposure to selective serotonin reuptake inhibitor (SSRI) antidepressants had sex-dependent effects on neonatal brain microstructure (67). Sexually dimorphic features of the human serotonergic system during the prenatal period were also evidenced by a positive correlation between placental and brain serotonin levels found only in male but not female human fetuses (65).

## 3 Serotonin system and its components in the human placenta

### 3.1 Localization of serotonin system components in the human placenta

**Figure 2:**
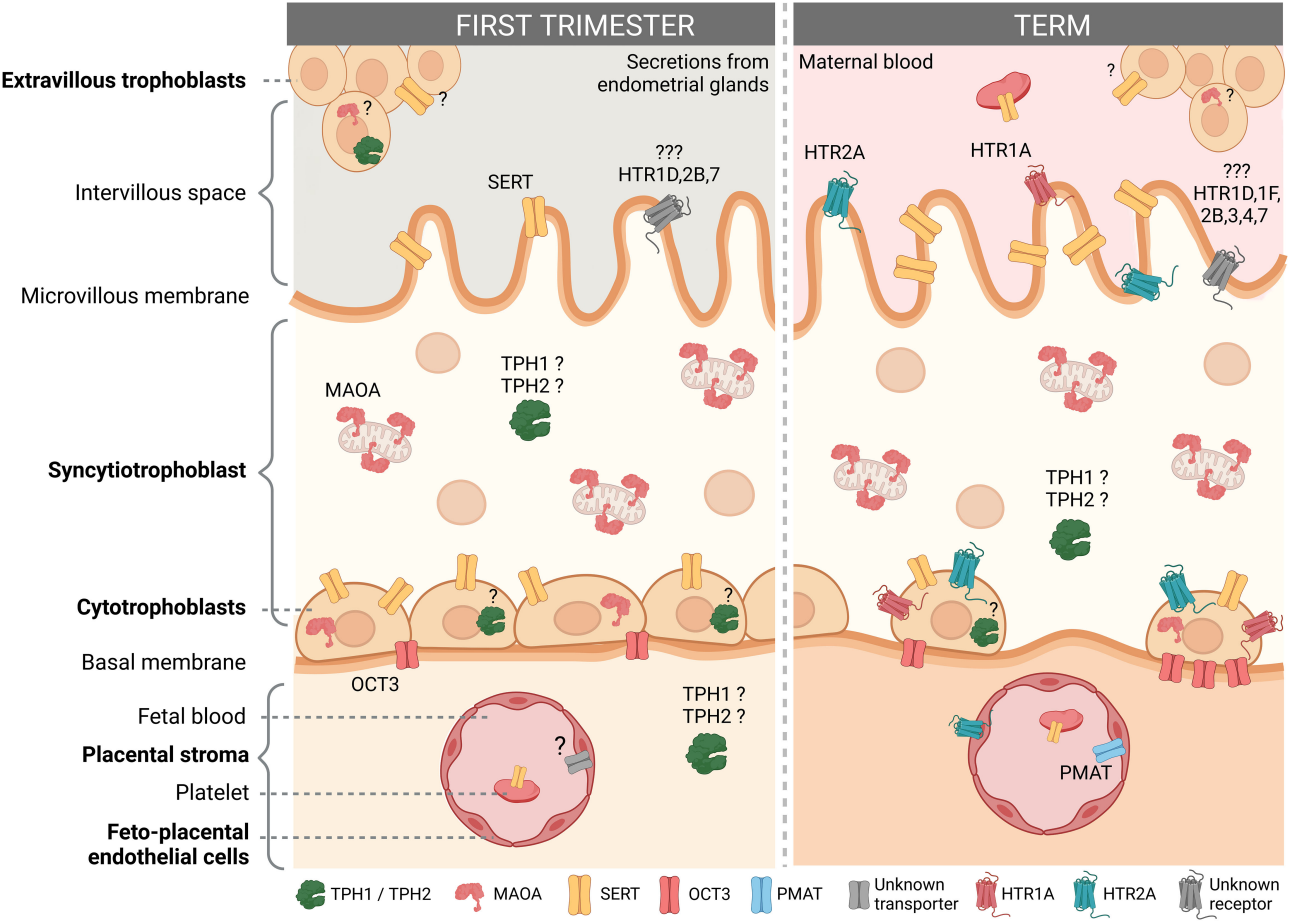
Localization of the serotonin system components in the human placenta according to gestational age. The figure shows the presence and localization of the components of the serotonin system in the human first trimester and term placenta according to the available literature. For details see main text. Components for which the results of different studies do not agree or which are reported only at the mRNA level are indicated with a question mark (?). Cell types not studied to date, such as mast cells, Hofbauer cells and other stromal cells are not shown. Created with BioRender.com.

The human placenta expresses many components of the serotonin system, including multiple serotonin receptor subtypes and several other proteins responsible for handling serotonin (**Figure 2**). The results of studies that examined the expression and/or localization of serotonin-related genes in human placenta are shown in **Table 1** (studies in placental tissue homogenates), **Table 2** (studies in placental tissue sections) and **Table 3** (studies in isolated placental cells). Several studies have also compared the expression of serotonin-related genes in the human placenta between different stages of pregnancy (**Table 4**).

**Table 1:** Presence of components of the serotonin system in homogenates of human placental tissue.

	Gestational period	mRNA	Protein	Activity	Study
**Enzymes**					
**TPH1**	First trimester	+ ^a^	*n.a.*	+ ^g^	(68)
	Term	+ ^a^	*n.a.*	+ ^g^	(68)
	First trimester	+ ^b^	(+) ^e^	+ ^g^	(69)
	Term	(+) ^b^	+ ^e^	+ ^g^	(69)
	Term	(+)/− ^a^	*n.a.*	*n.a.*	(70)
**TPH2**	First trimester	− ^a^	*n.a.*	+ ^g^	(68)
	Term	+ ^a^	*n.a.*	+ ^g^	(68)
	First trimester	(+) ^b^	*n.a.*	+ ^g^	(69)
	Term	(+) ^b^	*n.a.*	+ ^g^	(69)
	Term	(+)/− ^a^	*n.a.*	*n.a.*	(70)
**MAOA**	First trimester	+ ^b^	+ ^e^	+	(69)
	Term	+ ^b^	+ ^e^	+	(69)
	Term	+ ^b^	+ ^f^	+	(71)
	Term	+ ^b^	*n.a.*	*n.a.*	(72)
	Term	*n.a.*	+ ^h^	+	(73)
**MAOB**	Term	*n.a.*	− ^h^	−	(73)
	Term	+ ^b^	*n.a.*	*n.a.*	(71)
	Term	+ ^b^	*n.a.*	*n.a.*	(72)
**Transporters**					
**OCT1**	First trimester	+ ^a^	*n.a.*	*n.a.*	(74)
**(SLC22A1)**	Term	+ ^a^	*n.a.*	*n.a.*	(74)
	Term	(+) ^a^	*n.a.*	*n.a.*	(75)
	Term	− ^a^	*n.a.*	*n.a.*	(76)
	Term	− ^b^	*n.a.*	*n.a.*	(77)
**OCT2**	First trimester	+ ^a^	*n.a.*	*n.a.*	(74)
**(SLC22A2)**	Term	(+) ^a^	*n.a.*	*n.a.*	(74)
	Term	(+) ^a^	*n.a.*	*n.a.*	(75)
	Term	− ^a^	*n.a.*	*n.a.*	(76)
	Term	− ^b^	*n.a.*	*n.a.*	(77)
**OCT3**	First trimester	+ ^a^	*n.a.*	*n.a.*	(74)
**(SLC22A3)**	First trimester	(+) ^a^	(+) ^d^	*n.a.*	(75)
	Second trimester	+ ^a^	+ ^d^	*n.a.*	(75)
	Term	(+) ^a^	*n.a.*	*n.a.*	(74)
	Term	+ ^a^	+ ^d^	*n.a.*	(75)
	Term	+ ^a^	*n.a.*	*n.a.*	(76)
	Term	+ ^b^	*n.a.*	*n.a.*	(77)
**PMAT (SLC29A4)**	Term	(+) ^a^	*n.a.*	*n.a.*	(75)
**VMAT2 (SLC18A2)**	Term	(+) ^b^	*n.a.*	*n.a.*	(78)
**Receptors**					
**HTR2A**	Term	+ ^b^	+ ^e^	*n.a*	(79)
	Term	+ ^b^	+ ^e^	*n.a*	(80)
**HTR2B**	Term	+ ^a^	*n.a.*	*n.a.*	(81)
**HTR1D**	Term	+ ^a^	*n.a.*	*n.a.*	(68)
**HTR1E**	Term	+ ^a^	*n.a.*	*n.a.*	(68)
**HTR5A**	Term	+ ^a^	*n.a.*	*n.a.*	(68)
**HTR5B**	Term	+ ^a^	*n.a.*	*n.a.*	(68)

a RT−qPCR (quantitative reverse transcriptase−polymerase chain reaction).

b RT−PCR (reverse transcription−end point PCR).

c ddPCR (digital droplet PCR).

d LC−MS/MS (liquid chromatography coupled with tandem mass spectrometry).

e Western blot.

f ELISA (enzyme-linked immunosorbent assay).

g The activity assay used does not distinguish between the activity of TPH1 and TPH2.

h Binding studies with isoform-specific inhibitors.

n.a.: not analysed; **+**: detected; **−**: not detected; (+): low levels detected; (+)/−: low levels detected or below detection limit.

**Table 2:** Results on the localization of mRNAs and proteins of serotonin-related genes in human placenta obtained by *in situ* hybridization (ISH) and by immunohistochemical analysis (ISH), respectively.

	Gestational period	Location	mRNA/protein	Finding	Study
**Enzymes**					
**TPH1**	Weeks 7 to 39	All placental cells	protein	**−**	(82)
	First trimester	Syncytiotrophoblast	protein	**+**	(68, 83)
	First trimester	Cytotrophoblasts	protein	**+**	(68, 83)
	First trimester	Extravillous cytotrophoblasts	protein	**+**	(68, 83)
	First trimester	Villous stromal cells	protein	**+**	(68, 83)
	Term	Syncytiotrophoblast	protein	**+**	(68, 83)
	Term	Cytotrophoblasts	protein	**+**	(68, 83)
**TPH2**	First trimester	Syncytiotrophoblast	protein	**+**	(68, 83)
	First trimester	Cytotrophoblasts	protein	**+**	(68, 83)
	First trimester	Extravillous trophoblast	protein	**+**	(68, 83)
	First trimester	Villous stromal cells	protein	**+**	(68, 83)
	Term	Syncytiotrophoblast	protein	**+**	(68, 83)
	Term	Cytotrophoblasts	protein	**+**	(68, 83)
**MAOA**	Weeks 8, 12, 40	Syncytiotrophoblast	protein	**+**	(82)
	Weeks 8, 12, 40	Cytotrophoblasts	protein	**−**	(82)
	Term	Syncytiotrophoblast	protein	**+**	(84)
	Term	Syncytiotrophoblast	protein	**+**	(71)
	Term	Syncytiotrophoblast	mRNA	**+**	(72)
	Term	Cytotrophoblasts	mRNA	(+)	(72)
	Term	Vascular smooth muscle and endothelial cells	mRNA	(+)	(72)
**MAOB**	Term	Syncytiotrophoblast	protein	**−**	(71)
	Term	Syncytiotrophoblast	mRNA	**+**	(72)
	Term	Cytotrophoblasts	mRNA	**+**	(72)
	Term	Vascular smooth muscle and endothelial cells	mRNA	**+**	(72)
**Transporters**					
**SERT (SLC6A4)**	Weeks 7 to 41	Syncytiotrophoblast	protein	**+**	(82)
	Weeks 7 to 41	Cytotrophoblasts	protein	**+**	(82)
	Term	Syncytiotrophoblast	protein	**+**	(80)
	Term	Cytotrophoblasts	protein	**+**	(80)
	Term	Feto**−**placental endothelial cells	protein	**+**	(80)
**OCT1 (SLC22A1)**	Term	Intima layer of some placental vessels	mRNA	**−/+**	(78)
**OCT2 (SLC22A2)**	Term	Intima layer of some placental vessels	mRNA	**−/+**	(78)
**OCT3 (SLC22A3)**	Term	Adventitial cells of placental vessels	mRNA	**−/+**	(78)
	Term	Syncytiotrophoblast	protein	**+**	(85)
	Term	Feto-placental endothelial cells	protein	**+**	(85)
	Weeks 7 to 39	Syncytiotrophoblast	protein	**−**	(82)
	Weeks 7 to 39	Cytotrophoblasts	protein	**+**	(82)
	Weeks 7 to 39	Mesenchymal cells	protein	**+**	(82)
**VMAT2 (SLC18A2)**	Term	Extravillous cytotrophoblasts	mRNA	**−/+**	(78)
**Receptors**					
**HTR2A**	Term	Syncytiotrophoblast	protein	**+**	(80)
	Term	Cytotrophoblasts	protein	**+**	(80)
	Term	Feto**−**placental endothelial cells	protein	**+**	(80)
**HTR1A**	Term	Syncytiotrophoblast	mRNA	**+**	(86)
	Term	Cytotrophoblasts	mRNA	**+**	(86)
	Term	Syncytiotrophoblast	protein	+	(86)
	Term	Cytotrophoblasts	protein	**+**	(86)

**+**: detected; **−**: not detected; **−/+**: sporadically detected weak signal.

**Table 3:** Results on the presence of components of the serotonin system in human primary cell cultures (PCC) or single cells (SC).

	Gestational period	Cell type	PCC/SC	mRNA	Protein	Activity	Study
**Enzymes**							
**TPH1**	First trimester	Syncytiotrophoblast	PCC	**+** ^a^	*n.a.*	*n.a.*	(83)
	First trimester	Cytotrophoblasts	PCC	**+** ^a^	*n.a.*	*n.a.*	(83)
	First trimester	Extravillous cytotrophoblasts	PCC	**+** ^a^	*n.a.*	*n.a.*	(83)
	Term	Syncytiotrophoblast	PCC	**+** ^a^	+ ^e^	*n.a.*	(83)
	Term	Cytotrophoblasts	PCC	**+** ^a^	+ ^e^	*n.a.*	(83)
	Term	Syncytiotrophoblast	SC	**−** ^b^	*n.a.*	*n.a.*	(87)
	Term	Cytotrophoblasts	SC	**−** ^b^	*n.a.*	*n.a.*	(87)
	Term	Extravillous cytotrophoblasts	SC	**−** ^b^	*n.a.*	*n.a.*	(87)
	Term	Trophoblasts	PCC	*n.a.*	*n.a.*	**+** ^g^	(68)
**TPH2**	First trimester	Syncytiotrophoblast	PCC	**+** ^a^	*n.a.*	*n.a.*	(83)
	First trimester	Cytotrophoblasts	PCC	**+** ^a^	*n.a.*	*n.a.*	(83)
	First trimester	Extravillous cytotrophoblasts	PCC	**+** ^a^	*n.a.*	*n.a.*	(83)
	Term	Syncytiotrophoblast	PCC	**+** ^a^	+ ^e^	*n.a.*	(83)
	Term	Cytotrophoblasts	PCC	**+** ^a^	**+** ^e^	*n.a.*	(83)
	Term	Syncytiotrophoblast	SC	**−** ^b^	*n.a.*	*n.a.*	(87)
	Term	Cytotrophoblasts	SC	(+) ^b^	*n.a.*	*n.a.*	(87)
	Term	Extravillous cytotrophoblasts	SC	**−** ^b^	*n.a.*	*n.a.*	(87)
**MAOA**	First trimester	Syncytiotrophoblast	SC	+ ^b^	*n.a.*	*n.a.*	(88)
	First trimester	Cytotrophoblasts	SC	+ ^b^	*n.a.*	*n.a.*	(88)
	First trimester	Extravillous cytotrophoblasts	SC	+ ^b^	*n.a.*	*n.a.*	(88)
	Term	Syncytiotrophoblast	SC	+ ^b^	*n.a.*	*n.a.*	(87)
	Term	Cytotrophoblasts	SC	+ ^b^	*n.a.*	*n.a.*	(87)
	Term	Extravillous cytotrophoblasts	SC	+ ^b^	*n.a.*	*n.a.*	(87)
	Term	Trophoblasts	PCC	+ ^a^	*n.a.*	*n.a.*	(89)
	Term	Feto-placental endothelial cells	PCC	+ ^a^	*n.a.*	*n.a.*	(89)
**MAOB**	First trimester	Syncytiotrophoblast	SC	**−** ^b^	*n.a.*	*n.a.*	(88)
	First trimester	Cytotrophoblasts	SC	**−** ^b^	*n.a.*	*n.a.*	(88)
	First trimester	Extravillous cytotrophoblasts	SC	**−** ^b^	*n.a.*	*n.a.*	(88)
	Term	Syncytiotrophoblast	SC	**−** ^b^	*n.a.*	*n.a.*	(87)
	Term	Cytotrophoblasts	SC	**−** ^b^	*n.a.*	*n.a.*	(87)
	Term	Extravillous cytotrophoblasts	SC	**−** ^b^	*n.a.*	*n.a.*	(87)
**Transporters**							
**SERT**	First trimester	Syncytiotrophoblast	SC	+ ^b^	*n.a.*	*n.a.*	(88)
**(SLC6A4)**	First trimester	Cytotrophoblasts	SC	+ ^b^	*n.a.*	*n.a.*	(88)
	First trimester	Extravillous cytotrophoblasts	SC	(+) ^b^	*n.a.*	*n.a.*	(88)
	Term	Syncytiotrophoblast	SC	+ ^b^	*n.a.*	*n.a.*	(87)
	Term	Cytotrophoblasts	SC	+ ^b^	*n.a.*	*n.a.*	(87)
	Term	Extravillous cytotrophoblasts	SC	+ ^b^	*n.a.*	*n.a.*	(87)
	Term	Syncytiotrophoblast	PCC	+ ^c^	**+** ^e^	*n.a.*	(80)
	Term	Cytotrophoblasts	PCC	+ ^c^	**+** ^e^	*n.a.*	(80)
	Term	Feto**-**placental endothelial cells	PCC	(+) ^a^	*n.a.*	**−**	(89)
**OCT1**	Term	Trophoblasts	PCC	(+) ^a^	*n.a.*	*n.a.*	(89)
**(SLC22A1)**	Term	Feto**-**placental endothelial cells	PCC	(+)/− ^a^	*n.a.*	*n.a.*	(89)
**OCT2**	Term	Trophoblasts	PCC	**−** ^a^	*n.a.*	*n.a.*	(89)
**(SLC22A2)**	Term	Feto**-**placental endothelial cells	PCC	**−** ^a^	*n.a.*	*n.a.*	(89)
**OCT3**	First trimester	Syncytiotrophoblast	SC	**+** ^b^	*n.a.*	*n.a.*	(88)
**(SLC22A3)**	First trimester	Cytotrophoblasts	SC	**+** ^b^	*n.a.*	*n.a.*	(88)
	First trimester	Extravillous cytotrophoblasts	SC	**+** ^b^	*n.a.*	*n.a.*	(88)
	Term	Syncytiotrophoblast	SC	**+** ^b^	*n.a.*	*n.a.*	(87)
	Term	Cytotrophoblasts	SC	**−** ^b^	*n.a.*	*n.a.*	(87)
	Term	Extravillous cytotrophoblasts	SC	**−** ^b^	*n.a.*	*n.a.*	(87)
	Term	Trophoblasts	PCC	(+)/− ^a^	*n.a.*	*n.a.*	(89)
	Term	Feto**-**placental endothelial cells	PCC	(+)/− ^a^	*n.a.*	*n.a.*	(89)
**PMAT**	First trimester	Syncytiotrophoblast	SC	**−** ^b^	*n.a.*	*n.a.*	(88)
**(SLC29A4)**	First trimester	Cytotrophoblasts	SC	**−** ^b^	*n.a.*	*n.a.*	(88)
	First trimester	Extravillous cytotrophoblasts	SC	**−** ^b^	*n.a.*	*n.a.*	(88)
	Term	Syncytiotrophoblast	SC	**−** ^b^	*n.a.*	*n.a.*	(87)
	Term	Cytotrophoblasts	SC	**−** ^b^	*n.a.*	*n.a.*	(87)
	Term	Extravillous cytotrophoblasts	SC	**−** ^b^	*n.a.*	*n.a.*	(87)
	Term	Trophoblasts	PCC	**−** ^a^	*n.a.*	**−**	(89)
	Term	Feto**-**placental endothelial cells	PCC	**+** ^a^	*n.a.*	**+**	(89)
**VMAT1**	First trimester	Syncytiotrophoblast	SC	**−** ^b^	*n.a.*	*n.a.*	(88)
**(SLC18A1)**	First trimester	Cytotrophoblasts	SC	**−** ^b^	*n.a.*	*n.a.*	(88)
	First trimester	Extravillous cytotrophoblasts	SC	**−** ^b^	*n.a.*	*n.a.*	(88)
	Term	Syncytiotrophoblast	SC	**−** ^b^	*n.a.*	*n.a.*	(87)
	Term	Cytotrophoblasts	SC	**−** ^b^	*n.a.*	*n.a.*	(87)
	Term	Extravillous cytotrophoblasts	SC	**−** ^b^	*n.a.*	*n.a.*	(87)
**VMAT2**	Term	Trophoblasts	PCC	**−** ^d^	*n.a.*	**−**	(90)
**(SLC18A2)**	Term	Syncytiotrophoblast	SC	(+) ^b^	*n.a.*	*n.a.*	(87)
	Term	Cytotrophoblasts	SC	(+) ^b^	*n.a.*	*n.a.*	(87)
	Term	Extravillous cytotrophoblasts	SC	**+** ^b^	*n.a.*	*n.a.*	(87)
**Receptors**							
**HTR2A**	Term	Cytrophoblasts	PCC	**+** ^c^	**+** ^e^	*n.a.*	(80)
	Term	Syncytiotrophoblast	PCC	**+** ^c^	**+** ^e^	*n.a.*	(80)
	Term	Cytotrophoblasts	SC	(+) ^b^	*n.a.*	*n.a.*	(87)
	Term	Syncytiotrophoblast	SC	**−** ^b^	*n.a.*	*n.a.*	(87)
	Term	Extravillous cytotrophoblasts	SC	**−** ^b^	*n.a.*	*n.a.*	(87)
	Term	Trophoblasts	PCC	*n.a.*	**+** ^f^	*n.a.*	(91)
**HTR2B**	First trimester	Cytotrophoblasts	SC	**+** ^b^	*n.a.*	*n.a.*	(88)
	First trimester	Syncytiotrophoblast	SC	**−** ^b^	*n.a.*	*n.a.*	(88)
	First trimester	Extravillous cytotrophoblasts	SC	**−** ^b^	*n.a.*	*n.a.*	(88)
	Term	Cytotrophoblasts	SC	**+** ^b^	*n.a.*	*n.a.*	(87)
	Term	Syncytiotrophoblast	SC	**+** ^b^	*n.a.*	*n.a.*	(87)
	Term	Extravillous cytotrophoblasts	SC	**−** ^b^	*n.a.*	*n.a.*	(87)
**HTR1D**	Term	Syncytotrophoblast	SC	(+) ^b^	*n.a.*	*n.a.*	(87)
**HTR1F**	Term	Syncytotrophoblast	SC	(+) ^b^	*n.a.*	*n.a.*	(87)
**HTR3 (A)**	Term	Cytotrophoblasts	SC	(+) ^b^	*n.a.*	*n.a.*	(87)
**HTR4**	Term	Cytotrophoblasts	SC	(+) ^b^	*n.a.*	*n.a.*	(87)
**HTR7**	First trimester	Cytotrophoblasts	SC	**+** ^b^	*n.a.*	*n.a.*	(88)

a RT−qPCR (quantitative reverse transcriptase−polymerase chain reaction.

b RNA-sequencing.

c RT−PCR (reverse transcription−end point PCR).

d Northern blot.

e Western blot.

f Receptor binding studies.

g The activity assay used does not distinguish between the activity of TPH1 and TPH2.

n.a.: not analysed; **+**: detected; **−**: not detected; **(+)**: low levels detected; (+)/−: low levels detected or below detection limit.

**Table 4:** Changes in the expression of serotonin components in human placenta in the third trimester compared with the first trimester of pregnancy.

Gene	Location	mRNA/protein	First trimester	Term	Change*	Study
**Enzymes**						
TPH1	Tissue homogenate	mRNA	y	y	↓	(69)
	Tissue homogenate	protein	y	y	=	(69)
	Tissue homogenate	activity	y	y	=	(69)
TPH2	Tissue homogenate	mRNA	y	y	=	(69)
	Tissue homogenate	mRNA	n	y	↑	(68)
MAOA	Tissue homogenate	mRNA	y	y	↓	(69)
	Tissue homogenate	protein	y	y	=	(69)
	Tissue homogenate	activity	y	y	↑	(69)
	Syncytiotrophoblast	protein	y	y	=	(82)
MAOB	Cytotrophoblasts	mRNA	n	y	↑	(87, 88)
**Transporters**						
SERT	Tissue homogenate	mRNA	y	y	↓	(69)
(SLC6A4)		protein	y	y	↑	(69)
OCT1	Tissue homogenate	mRNA	y	y	=	(74)
(SLC22A1)						
OCT2	Tissue homogenate	mRNA	y	y	↓	(74)
(SLC22A2)						
OCT3	Tissue homogenate	mRNA	y	y	=	(69, 75)
(SLC22A3)	Tissue homogenate	protein	y	y	↑	(69, 75)
	Tissue homogenate	mRNA	y	y	↓	(74)
	Cytotrophoblasts	mRNA	y	n	↓	(87, 88)
	Extravillous cytotrophoblasts	mRNA	y	n	↓	(87, 88)
PMAT	Cytotrophoblasts	mRNA	y	n	↓	(87, 88)
(SLC29A4)						
**Receptors**						
HTR1D	Cytotrophoblasts	mRNA	y	n	↓	(87, 88)
	Syncytiotrophoblast	mRNA	n	y	↑	(87, 88)
	Extravillous cytotrophoblasts	mRNA	y	n	↓	(87, 88)
HTR2A	Cytotrophoblasts	mRNA	n	y	↑	(87, 88)
HTR2B	Syncytiotrophoblast	mRNA	n	y	↑	(87, 88)
HTR7	Cytotrophoblasts	mRNA	y	n	↓	(87, 88)

y: detected, n: not detected.

* ↑, up-regulated; ↓, down-regulated; =, no change at term of pregnancy compared to first trimester.

#### 3.1.1 Serotonin

Evidence on the presence of serotonin itself in the human placental cells is yet inconclusive. An early immunohistochemical (IHC) study of the human term placenta reported the presence of serotonin in syncytiotrophoblast, stromal cells, and capillary endothelium (86). However, recent IHC study of the human term placenta detected serotonin only in platelets in the chorionic villus vessels and maternal intervillous space, but not in untreated syncytiotrophoblast and cytotrophoblasts (82). Similarly, in the human first and second trimester placentas, platelets were strongly stained for serotonin, while only traces of serotonin were seen in untreated trophoblast cells (82). In the presence of exogenously added serotonin, both cytoplasmic and nuclear compartments of the cytotrophoblast stained for serotonin, but only nuclei in the syncytiotrophoblast stained for serotonin, whereas cytoplasmic serotonin staining in the syncytiotrophoblast was observed only after pharmacological blockade of serotonin catabolism (82). This suggests that serotonin levels in the syncytiotrophoblast are tightly controlled by rapid enzymatic degradation.

#### 3.1.2 Serotonin-synthesizing enzymes

Studies also disagree about the presence of serotonin synthesis and expression of serotonin-synthesizing enzymes in the placenta. It has long been assumed that all serotonin affecting peripheral organ functions is synthesized in the intestine and distributed throughout the body by circulating platelets (**Figure 1**). This classical view of serotonin as a gut-derived hormone has been extended by the discovery of local sources of serotonin in various organs expressing TPH1, a peripheral isoform of the rate-limiting enzyme in serotonin synthesis (26). As for the placenta, an initial study demonstrated a lack of serotonin synthesis in the mouse placenta (92). However, Bonnin et al. (93) found evidence for serotonin synthesis in both mouse and human placenta. They demonstrated that levels of both serotonin and its immediate precursor (5-HTP) increase in homogenates of human first trimester placenta incubated with L-tryptophan (L-trp) and tetrahydrobiopterin (BH4, cofactor for TPH1 and TPH2 activity) (93). Conversion of L-trp to 5-HTP or serotonin in homogenates of human first trimester placenta has been replicated in independent studies and has also been demonstrated in homogenates of human term placenta (68, 69) and in cultured human term syncytiotrophoblast (83).

In addition, the presence of *TPH1* and *TPH2* mRNAs (encoding the peripheral and neuronal isoforms, respectively, of the rate-limiting enzyme of serotonin synthesis) and the presence of TPH1 and TPH2 proteins was demonstrated in primary syncytiotrophoblast and cytotrophoblasts isolated from human first trimester and term placentas (83). Studies in human placental homogenates reported that *TPH1* mRNA was the predominant isoform throughout pregnancy (69) or the only one detected in the first trimester (68). In IHC analysis, TPH1 and TPH2 proteins were localized in syncytiotrophoblast, cytotrophoblasts, and some stromal cells of human first trimester and term placentas (68, 83).

In contrast to the above results, another IHC study failed to find TPH1 protein in human placentas from the first and second trimesters of pregnancy, while rare TPH1 signals were observed in human term placentas, differing from strong signals in human appendix (82). In addition, single-cell transcriptome analysis by RNA-sequencing (RNA-seq) did not detect *TPH1* mRNAs in human term syncytiotrophoblast and cytotrophoblasts, whereas *TPH2* mRNAs were detected only in term cytotrophoblasts, but at very low levels (87).

#### 3.1.3 Serotonin-catabolizing enzymes

Serotonin catabolizing isoenzyme MAOA is abundant in the human placenta throughout pregnancy, as demonstrated by IHC (82) and enzyme activity studies (69). Known sites of *MAOA* expression in human placenta appear to be syncytiotrophoblast (29, 72, 82, 84), cytotrophoblasts (72, 87, 88) and feto-placental endothelial cells (89). Single cell transcriptomic data show that syncytiotrophoblast as compared with cytotrophoblasts contain much higher levels of *MAOA* mRNA (about 10-fold higher in the first trimester, and about 2-fold higher at term) (87, 88). mRNA encoding MAOB, MAO isoform with a lower affinity for serotonin, was detected at very low levels in term placenta (70, 87) and was absent in first trimester placenta (88).

#### 3.1.4 Serotonin receptors

Ten serotonin receptor subtypes (HTR1A, HTR1D, HTR1E, HTR1F, HTR2A, HTR2B, HTR3 (subunit HTR3A), HTR4, HTR5A and HTR5B) have been reported to be expressed in the human term placenta (68, 79–81, 86, 87, 91). HTR2A has been localized in syncytiotrophoblast, cytotrophoblasts and fetal capillary endothelium of human term placentas (80). HTR2B, the most abundant subtype according to single cell transcriptomic analyses, is also expressed in both syncytiotrophoblast and cytotrophoblasts (81, 87). In contrast, *HTR1D* and *HTR1F* mRNAs were detected only in syncytiotrophoblast, whereas *HTR3A* and *HTR4* mRNAs were found at low levels only in cytotrophoblasts (87) of human term placentas. So far, only *HTR1D*, *HTR2B* and *HTR7* mRNAs have been detected in human first trimester placentas (specifically in cytotrophoblasts) (88).

However, it should be emphasized that the expression of most serotonin receptor subtypes in human placenta (with the exception of HTR2A (79, 80, 91) and HTR1A (86)) has been detected only at the mRNA level, so the presence and location of their proteins and their functions in human placenta require further investigation.

#### 3.1.5 Vesicular transporters

Transcripts encoding VMAT2 were not detected in cultured human term trophoblasts by Northern blot analysis, and functional analysis demonstrated the absence of VMAT activity in the human trophoblast cell line JAR (90). Furthermore, *in situ* hybridization (ISH) did not detect *VMAT2* mRNA in human villous trophoblasts, while only a weak signal was occasionally seen in extravillous trophoblasts within the uterine wall; very low levels were detected in placental homogenates by the more sensitive RT-PCR analysis (78), which may also have targeted platelet mRNA content. Single-cell transcriptome (RNA-seq) analyses reported that *VMAT1* mRNA was not found in trophoblasts throughout pregnancy, while only very low levels of *VMAT2* mRNA were detected in term trophoblasts (87, 88). Taken together, these results indicate that serotonin in trophoblasts is not stored in intracellular vesicles.

#### 3.1.6 Plasma membrane transporters

Human placenta expresses several plasma membrane transporters for serotonin. Activity of the high-affinity SERT has been shown in plasma membrane vesicles (70, 94) and primary trophoblasts (89) isolated from human term placenta. *SERT* mRNA levels were lower in term compared to first trimester placentas, while the opposite was found for SERT protein levels (69). IHC analyses localized SERT protein to both syncytiotrophoblast and cytotrophoblasts (80, 82). Single-cell transcriptome analyses showed lower *SERT* mRNA levels in syncytiotrophoblast and cytotrophoblasts, both in the first trimester (88) and at term (87). On the other hand, recent study found that *SERT* mRNA levels were upregulated during spontaneous syncytialization of human primary trophoblasts, but SERT protein and activity levels were downregulated by syncytialization (95). Low levels of *SERT* mRNA and SERT protein were also detected in feto-placental endothelial cells (80, 82, 89), but functional analysis did not support significant SERT activity in these cells (89).

Low-affinity, polyspecific transporters OCT1 and OCT2 have been reported to be absent (77) or expressed at very low levels (74–76, 78, 89) in human first trimester and term placentas. This is consistent with the absence of their mRNAs in single cell transcriptome analyses (87, 88).

OCT3 is the most prominent member of OCT family and abundant in the human placenta throughout pregnancy (76, 77), with slightly lower mRNA and protein levels found in the first trimester than at term (69, 75). OCT3 protein and activity were detected in membrane vesicles isolated from the fetus-facing (basal) side, but not from the maternal-facing (microvillous) side of the human term placenta (70, 77). However, the exact cell type(s) harboring this transporter are not entirely clear. Thus, in one IHC study, prominent OCT3 staining was found on basolateral surface and in cytoplasm of cytotrophoblasts, while it was absent in syncytiotrophoblast in all three trimesters of pregnancy (82). This is consistent with OCT3 protein levels being down-regulated during spontaneous syncytialization of human term primary trophoblasts (95). However, in another study, OCT3 was localized to the basal (fetal-facing), but not the apical (maternal-facing) membrane of term syncytiotrophoblast (85). Weak OCT3 staining was occasionally observed in fetal capillaries (82, 85), but *OCT3* mRNA was not detected in primary feto-placental endothelial cells (89). Single-cell transcriptome analysis by RNA-Seq demonstrated weak *OCT3* mRNA signal in all types of first trimester trophoblasts (88), while at the end of pregnancy, *OCT3* transcripts were detected only in syncytiotrophoblast (87).

Low levels of *PMAT* mRNA, encoding another low-affinity serotonin transporter, were detected in human term placental tissue (75). *PMAT* mRNA was absent in human first trimester (88) and term trophoblasts (87, 89), but was detected in human term feto-placental endothelial cells (89). In addition, efficient low-affinity serotonin uptake activity was detected in feto-placental endothelial cells, most likely mediated by PMAT (89).

### 3.2 Functions of serotonin system components in the human placenta

Serotonin is a potent vasoactive agent. Therefore, it was recognized early on that the placental serotonin system may contribute to the regulation of umbilico-placental blood flow. Serotonin induced a strong contractile effect on human umbilical and placental arteries and veins in tissue explants (96–98). Ketanserin, a known antagonist of the HTR2A receptor, significantly decreased the contractile response to serotonin in chorionic artery and vein segments from human placentas, suggesting that HTR2A is likely the receptor subtype mediating the vasoconstrictive effects of serotonin (98). At the cellular level, several studies have aimed to understanding the function and regulation of the serotonin system in the human placenta using cell culture models. Some of these studies have been performed on the human trophoblast-like cell lines such as BeWo, JEG-3 and JAR. These choriocarcinoma-derived cells are commonly used *in vitro* models in human placenta research (99). Recently, however, concerns have been raised about their suitability for studying serotonin pathways (70, 89, 95). Suitable *in vitro* models for future studies would be primary trophoblasts, placental explants, and organoid trophoblast cultures (100), as well as primary feto-placental endothelial cells, Hofbauer cells, and other cell types. Also, *in vitro* studies should preferably be conducted at physiological oxygen tension as *TPH1* and *SERT* have been shown to be oxygen-regulated in pulmonary endothelial and smooth muscle cells, respectively (101, 102).

Thus, serotonin has been shown to stimulate proliferation of human placental trophoblast-like cell lines (BeWo, JEG-3) through activation of the HTR2A receptor and subsequent activation of a downstream signaling cascade involving both the ERK1/2 and STAT3 signaling pathways (79, 103, 104). Based on the known roles of the ERK1/2 and STAT3 signaling pathways, it has been proposed that serotonin regulates placental development and structure by controlling cell viability, differentiation, migration and invasion (103, 104). *In vivo*, the role of serotonin in regulating placental development is supported by a study in a knock-out mouse model showing that altered serotonin levels in placental intervillous space impair trophoblast survival and disrupt normal placental structure (105).

One more role assigned to the HTR2A receptor in the human placenta relates to the regulation of placental estrogen production. Specifically, studies in human primary trophoblasts (106) as well as trophoblast-like cell lines (BeWo, JEG-3) (107) have shown that serotonin induces the expression and activity of aromatase CYP19, a key enzyme in placental estrogen synthesis, *via* activation of the HTR2A receptor. The observed increase in aromatase activity was induced by HTR2A-mediated stimulation of the protein kinase C pathway (107) and possibly the JAK2/STAT3 pathway (104).

The expression of various serotonin receptor subtypes (cf. 3.1.4), coupled to different intracellular signaling pathways, suggests diverse and as yet unknown roles for serotonin in the human placenta. For example, some of the serotonin receptor subtypes expressed in the human term placenta (i.e., HTR1F, HTR3, and HTR4) have been shown to regulate mitochondrial function and homeostasis in different mouse organs (108, 109). It would be interesting to investigate whether serotonin plays a similar role in the human placenta during late pregnancy.

### 3.3 Regulation of serotonin system components in the human placenta

The mechanisms that regulate components of the serotonin system in the human placenta are largely unexplored, with studies to date focusing on SERT and MAOA.

Expression of the *SERT* is regulated by two distinct variable number tandem repeat polymorphisms in the promoter and intron 2 region (5HTTLPR and STin2, respectively), as well as by several single nucleotide polymorphisms (SNPs) in the promoter region, including rs25531 and rs25532 (110). In addition to genetic variants, epigenetic mechanisms play an important role in the regulation of *SERT* expression. *SERT* promoter contains a region enriched in CpG dinucleotides (CpG island), a sequence context in which cytosine is frequently methylated. Increased methylation of this region correlates with decreased *SERT* mRNA expression (111). We have found that *SERT* mRNA levels in the human term placenta are predominantly determined by *SERT* methylation in this region and not by *SERT* genetic variants (45). Studies in the trophoblast-like cell line JAR suggest that small noncoding RNAs, namely miR-15a and miR-16, also play an important role in the epigenetic regulation of *SERT* expression in the human placenta (46).

Expression and activity of the *SERT* in JAR cells is modulated by cytokines. Interleukin-1 upregulated *SERT* expression in JAR cells *via* cyclic adenosine monophosphate-independent signaling pathways (112), whereas interleukin-6 downregulated its expression and activity *via* a STAT3-dependent signaling pathway (113).

SERT activity in placental cells is regulated also by hormones such as insulin and estrogen. Insulin upregulates SERT activity in primary human trophoblast by enhancing dissociation of SERT protein from the endoplasmic reticulum chaperone ERp44, thereby enabling its maturation and translocation to the cell surface (55). Estrogen (17β-estradiol) decreased the activity of SERT in trophoblast-like BeWo cells, but increased the level of SERT protein and had no effect on the level of *SERT* mRNA (114). A discrepancy between the levels of *SERT* mRNA and SERT protein in human placenta was also observed in relation to the effects of gestational age (69) and trophoblast differentiation (95), indicating the presence of important regulatory mechanisms acting at the translational level.

The increasing use of SERT-targeting antidepressants (115–117) and psychostimulants (118) during pregnancy has led to investigations into the potential of these drugs to alter SERT activity in the placenta. We have shown that many common antidepressants at therapeutic plasma concentrations effectively inhibit the activity of SERT in primary trophoblasts isolated from human term placentas (89). This finding and studies in other placental models (82, 94, 119–121) highlight that antidepressant therapy in pregnancy may affect serotonin homeostasis in the placenta.

Expression of the *MAOA* gene is regulated by a variable number tandem repeat polymorphism located upstream of the *MAOA* coding region (MAOA*-*uVNTR), consisting of a 30-bp sequence present in 2, 3, 3.5, 4 or 5 copies (122). In trophoblast-like JAR cells, alleles with 3.5 and 4 repeats were transcribed more efficiently than those with 3 or 5 repeats (122), suggesting that alleles with 3.5 and 4 repeats correspond to the optimal length of this regulatory region. Consistent with this, the 4-repeat allele was associated with higher levels of *MAOA* mRNA in human term placenta than the 3-repeat allele (123). The *MAOA* promoter also contains two CpG islands (124), but their role in regulating *MAOA* transcription in the placenta has not yet been studied.

### 3.4 Changes of placental serotonin system components in pregnancy pathologies

It has long been known that serotonin signaling plays a role in the pathogenesis of pre-eclampsia (PE), a hypertensive pregnancy disorder characterized by multiple organ dysfunction. As suggested recently (125), dysregulation of serotonin signaling may underlie several features of PE, including excessive platelet aggregation, vascular hyporeactivity, and pro-inflammation. The placentas of pregnancies complicated with PE show decreased MAOA activity (71, 126), which may lead to decreased serotonin catabolism and contribute to the elevated circulating serotonin levels observed in women with PE. On the other hand, placental SERT activity is unchanged in PE (126). Decreased vascular reactivity of placental and umbilical vessels to serotonin has also been noted in PE (96, 98), possibly contributing to decreased umbilico-placental blood flow (127).

Changes in placental serotonin system have been found also in gestational diabetes mellitus (GDM), with most studies focusing on SERT. An initial study in a small cohort found decreased *SERT* mRNA and protein levels in placentas from GDM pregnancies (80). However, later studies with larger and better defined samples reported increased *SERT* mRNA levels in GDM placentas (45, 128). In contrast, primary trophoblasts isolated from GDM placentas showed decreased activity of SERT, which was attributed to decreased localization of the SERT protein to the plasma membrane (55). Interestingly, trophoblasts isolated from GDM placentas also showed an attenuated response to *in vitro* insulin treatment in terms of dissociation of SERT from the endoplasmic reticulum chaperone ERp44, suggesting that defects in insulin signaling are responsible for the impaired functional expression of SERT on the cell surface in GDM trophoblasts (55). In addition to increased expression of *SERT*, GDM placentas exhibited decreased methylation of the *SERT* promoter region (45). It may be speculated that epigenetic mechanisms increase *SERT* transcription to counteract the impact of defective insulin signaling on the activity of SERT in GDM placentas. It has also been reported that expression of serotonin receptor HTR2A is decreased in GDM placentas (80). Interestingly, DNA methylation of the placental *HTR2A* gene was associated with maternal overweight/obesity and GDM only in female, but not in male placentas (129).

Maternal mental health in pregnancy was also associated with alterations in placental serotonin homeostasis. As with PE, downregulation of *MAOA* expression in the placenta has been associated with increased symptoms of maternal depression during pregnancy (130). Maternal alcohol consumption during pregnancy was associated with decreased *SERT* mRNA levels and increased *TPH1* mRNA levels in the placenta (131).

Recent evidence links alterations in the placental serotonin system to fetal growth restriction (FGR) (68) and preterm birth (132), serious pregnancy conditions accounting for significant perinatal morbidity and mortality. Thus, increased TPH activity and altered expression of *SERT*, *TPH2*, *HTR1D*, and *HTR5A* genes were found in third-trimester placentas from FGR pregnancies compared with gestational age-matched control pregnancies (68). In placentas from spontaneous preterm births, expression of both *SERT* and *OCT3* transporters was upregulated compared with placentas from term births (132).

In summary, changes of serotonin system in the human placenta have been associated with several pregnancy-related medical conditions, including PE, GDM, maternal overweight/obesity, maternal mental health in pregnancy, FGR, and preterm birth. Whether the observed changes are causally involved in the pathogenesis of the above pathologies or are their consequence, or both, requires further investigation. Further studies are also needed to validate these findings in independent samples and to investigate the mechanisms underlying the observed changes.

## 4 Outlook - future perspectives

### 4.1 Does the human placenta provide serotonin to the fetus?

An important role attributed to the placental serotonin system in animal studies is to provide the embryo/fetus with an exogenous source of serotonin needed for proper brain and other organ development (93, 133, 134). However, studies disagree on the exact source of serotonin delivered to the fetus *via* the placenta, supporting either its maternal origin (133) or its synthesis in the placenta (93). In humans, it is generally believed that the placenta supplies maternal and/or placental serotonin to the developing fetal brain until the late first/early second trimester of pregnancy, when serotonergic neurons organize in the raphe nuclei and synthesis begins *in situ* (19, 135). This is supported by a recent discovery of a (male-specific) correlation between serotonin levels in the human fetal brain and the placenta (65).

As mentioned earlier (cf. 3.1.2), most studies to date support the ability of the human placenta to synthesize serotonin during both early and late pregnancy (68, 69, 83, 93). However, it should be noted that none of these studies examined the time and substrate concentration dependence of 5-HTP or serotonin production, or applied pharmacological approaches to demonstrate the involvement of TPH1/2 enzyme(s) (68, 69, 83, 93). In addition, the TPH activity assays used relatively high concentrations of L-trp (200 or 250 µM) compared with the Michaelis-Menten constant of human TPH for L-trp (7.5 µM) (136). Thus, convincing evidence for serotonin synthesis in the human placenta is still lacking. This is also supported by contrasting results obtained with a combination of immunohistochemical and pharmacological experiments on human placental tissue sections and placental explants (82). These argue that serotonin synthesis does not occur in the human placenta at any stage of pregnancy. Rather, the results suggest that serotonin released from maternal platelets into the intervillous space may be taken up into the syncytiotrophoblast *via* SERT and subsequently transferred to the fetal blood *via* a putative pathway involving several other proteins such as gap junction connexin-43 and OCT3 (82).

Placental perfusion is a method allowing studies into potential transfer of substances from the maternal to the fetal side of the placenta or in the opposite direction. The possible release of substances synthesized in the placenta into the maternal or fetal circulation can also be studied by this method. The only perfusion study performed to date on the human placenta suggests that serotonin is not transferred in appreciable amounts from the maternal to the fetal side at term pregnancy (137). This is consistent with results showing that serotonin taken up in the human term syncytiotrophoblast is rapidly catabolized by MAOA (70). Perfusion studies investigating the transfer of maternal/placental serotonin into fetal blood during early human pregnancy have not yet been performed.

In conclusion, current evidence does not support transplacental transfer of maternal serotonin into the fetal circulation at the end of human pregnancy. Further studies are needed to determine whether this might be different at earlier stages of pregnancy and to clarify whether the human placenta is capable of synthesizing serotonin under physiological conditions.

### 4.2 Epigenetic regulation of serotonin system components and epigenetic effects of serotonin

Epigenetic mechanisms are central to gene regulation and gene-environment interactions and are therefore of particular importance to the biology of the placenta as an organ that responds to changes in the intrauterine environment (138). The regulation of placental serotonin homeostasis by epigenetic mechanisms remains largely unexplored – so far, only two studies have addressed the role of DNA methylation (45) and noncoding RNAs (46) in the regulation of serotonin-related genes in the human placenta. Further studies on the role of DNA methylation, histone modifications, and noncoding RNAs and how they are modulated by various factors are warranted. In addition, further important studies should encompass the interaction of epigenetic mechanisms with functionally relevant genetic polymorphisms and post-translational regulatory mechanisms.

In addition to investigating the epigenetic regulation of genes involved in serotonin signaling, also the possible role of serotonin as a regulator of placental gene expression warrants studies. Recently, serotonylation of glutamine 5 on histone H3 (H3Q5ser) was identified as an epigenetic mechanism involved in the regulation of genes important for neuronal cell differentiation (23). The H3Q5ser modification was found in both neuronal and non-neuronal brain cells as well as in heart, colon, and blood samples. Its presence in the placenta has not been examined. However, findings on the presence of serotonin in the nuclei of various human placental cells (82) encourage future research on its presence and role in the human placenta.

### 4.3 Role of placental serotonin system in the developmental origins of health and disease

Animal studies show that changes in maternal and placental serotonin homeostasis not only impact the developing fetal brain but also have lasting neurochemical and neurobehavioral consequences (113, 114, 116, 117). In humans, changes in serotonin levels in maternal blood have been linked to an increased risk of neurodevelopmental disorders in the offspring, such as attention deficit/hyperactivity disorder (139) and autism (140). As for the human placenta, methylation of the placental *HTR2A* gene has been linked to key behavioral measures of neurodevelopment (quality of movement and attention) in human newborns (141), and placental expression of the *SERT* gene has been linked to regulatory behaviors in infants at two weeks of age (142). Furthermore, placental expression of the *SERT* and *HTR2B* genes has been associated with the incidence of febrile seizure in children (81), while placental expression of the *MAOA* gene has been identified as a biological mediator of the association between prenatal stress and child temperament at 12 months of age (143). Further longitudinal studies in human cohorts are needed to better understand the consequences of altered placental serotonin homeostasis in the developmental origins of health and disease. As mentioned earlier, changes in placental serotonin homeostasis have been associated with various pregnancy disorders (cf. 3.4). It is, therefore, of utmost importance to clarify whether they are innocent bystanders or play a role in the development of the disorder or in mediating the consequences for fetal health outcomes. This could easily open up new therapeutic opportunities, as there are already many approved drugs available that modulate the activity of various serotonin system components (144–146).

### 4.4 The importance of fetal sex

A well-established feature of the serotonin system in the brain of both humans and rodents is sexual dimorphism (cf. 2). Studies in rodents showing that serotonin uptake into placental membrane vesicles differs between female and male fetuses (70) suggest a sexual dimorphism also in placental handling of serotonin. The sexually dimorphic nature of the serotonin system in the human placenta is supported by the male-specific correlation of serotonin levels in placenta and fetal brain (65). In addition, methylation of the *HTR2A* gene in human placenta has been shown to be associated with maternal overweight/obesity only in female placentas (129), suggesting that fetal sex modulates the sensitivity of the placental serotonin system to the intrauterine environment. Furthermore, studies in animals and humans suggest an interaction between sex hormones and the serotonin system. For example, estrogens modulate the synthesis, uptake and catabolism of serotonin (147), while changes in serotonin signaling have been reported to affect estrogen production (106, 148). Overall, the influence of fetal sex needs to be considered in future studies of the function, regulation, and pathology-related changes of the serotonin system in the human placenta.

## 5 Final comments

Phylogenetically, serotonin is an ancient molecule. However, it has taken a long while until the components of the complex system regulating activity of serotonin have been identified in general. Only in the past decade, the serotonin system has received attention for its potential role in reproduction and development. Studies into serotonin’s involvement in the maternal-fetal interplay to ultimately contribute to fetal development through its actions on and within the placenta have been hampered for a variety of reasons. These include, but are not limited to, availability of proper experimental systems that fully capture the complexity of cellular interplay in the placenta, the difficulty in separating potential effects of maternal from those of fetal serotonin and by the temporal changes of placental cellular composition and function throughout pregnancy.

In this review we have not only summarized the current knowledge in this emerging field, but also provided suggestions for further studies. We hope that new molecular and cellular data can be integrated into a larger framework to better understand the role of serotonin system for placental and fetal development and its potential dysregulation in pathological pregnancy conditions.

## Author contributions

JŠ and GD conceived and designed the article. MP, IB and JŠ wrote the first draft. MP and IB prepared the figures and tables. LČ-Š, GD, and JŠ critically revised the manuscript. All authors read and approved the final version.

## Funding

This work was funded by Croatian Science Foundation grant IP-2018-01-6547 to JŠ. MP is supported by Croatian Science Foundation grant DOK-2018-09-7794.

## Conflict of interest

The authors declare that the research was conducted in the absence of any commercial or financial relationships that could be construed as a potential conflict of interest.

## Publisher’s note

All claims expressed in this article are solely those of the authors and do not necessarily represent those of their affiliated organizations, or those of the publisher, the editors and the reviewers. Any product that may be evaluated in this article, or claim that may be made by its manufacturer, is not guaranteed or endorsed by the publisher.

## Abbreviations

5-HIAA, 5-hydroxyindoleacetic acid; 5-HT, 5-hydroxytryptamine; 5-HTP, 5-hydroxytryptophan; 5-HTR, 5-hydroxytryptamine receptor; BH4, tetrahydrobiopterin; FGR, fetal growth restriction; GDM, gestational diabetes mellitus; IHC, immunohistochemistry; L-trp, L-tryptophan; MAO A/B, monoamine oxidase A/B; PE, pre-eclampsia; OCT 1/2/3, organic cation transporter 1/2/3; PMAT, plasma membrane monoamine transporter; VMAT 1/2, vesicular monoamine transporter 1/2; SERT, serotonin transporter; TPH1/2, tryptophan hydroxylase 1/2.
